# Systemic and ocular diseases associated with the development of diabetic macular edema among Japanese patients with diabetes mellitus

**DOI:** 10.1186/s12886-020-01578-8

**Published:** 2020-07-29

**Authors:** Atsuki Kume, Kenji Kashiwagi

**Affiliations:** Department of Ophthalmology, University of Yamanashi, Chuo, Yamanashi Japan

**Keywords:** Diabetic macular edema, Diabetic mellitus, Risk factors, Claim database, International classification of diseases 10th revision

## Abstract

**Background:**

Diabetic macular edema (DME) causes severe vision loss among patients with diabetes mellitus (DM). We aimed to investigate systemic and ocular diseases associated with the development of DME in a Japanese population.

**Methods:**

A total of 3.11 million Japanese subjects who were registered in the database of the Japan Medical Data Center from 2005 to 2014 were analyzed. Subjects with DM were defined as individuals who had been prescribed any therapeutic medications for DM, and associated diseases were analyzed. The periods assessed were one year before the development of DME among patients with DME and one year before the last visit to an ophthalmic clinic among patients without DME.

**Results:**

A total of 17,403 patients with DM satisfied the inclusion and exclusion criteria, and 420 patients developed DME. Univariate analysis revealed significant associations between 55 diseases, including 39 systemic and 16 ocular diseases, and DME development. Logistic analysis identified 21 systemic diseases and 10 ocular diseases as significant factors associated with DME development.

**Conclusion:**

Various types of systemic and ocular diseases are associated with DME development. Subjects with DM who present these risk factors must be carefully monitored to prevent visual impairment.

## Background

The prevalence of diabetes mellitus (DM) continues to increase worldwide, and diabetic retinopathy (DR) remains a leading cause of vision loss in many countries [1, 2]. The reported prevalence of DR among patients with DM varies widely, from 1 to 40% [3–9]. According to Yau et al., approximately 93 million people worldwide develop DR, 17 million are diagnosed with proliferative DR (PDR), and 21 million are diagnosed with diabetic macular edema (DME) [10].

Although PDR is the most common vision-threatening retinopathy, DME is responsible for most of the vision loss experienced by patients with DM and remains the major cause of vision loss among patients with DM with or without PDR. Many previous population-based or hospital-based studies have focused on the prevalence of DME, yet there are few large-scale studies examining the incidence of DME. In addition, the annual incidence rates of DME in previous studies varied widely from 0.01 to 6.0% [11–15].

Previous epidemiological studies have also identified several risk factors associated with DR, including many systemic and lifestyle factors, nephropathy, obesity, alcohol consumption, hematological markers of anemia, hypothyroidism, inflammation, endothelial dysfunction, hyperglycemia, hypertension, dyslipidemia, diabetes duration, ethnic origin, pregnancy, and puberty [10, 16–21]. Some risk factors have been associated with DME development, including duration of diabetes, hemoglobin A1c, blood pressure, nephropathy, higher cholesterol, retinal and vitreous inflammation, and oxidative stress in the retina [22], but inconsistencies exist among reports [23]. Furthermore, risk factors for DME may differ from those for DR, and the precise roles of these factors in the pathogenesis of DME are not well defined. Some epidemiological studies have examined the prevalence, incidence, and risk factors for DM and DR, including studies employing the Japan Diabetes Clinical Data Management Study Group (JDDM). Unfortunately, an insufficient number of studies have focused on DME in Japan. Currently, the main therapeutic procedures for DME are laser photocoagulation, vitrectomy, and anti-vascular endothelial growth factor (VEGF) injections, though these approaches are not always effective. In addition to the three approaches mentioned above, targeted therapies can ameliorate some of the identified risk factors. Here, we investigate systemic and ocular factors associated with DME development in a large-scale study based on the health insurance claim database in Japan.

## Methods

This study was performed in accordance with the Declaration of Helsinki and was approved by the Institutional Ethics Review Board (IRB) of the University of Yamanashi. The IRB approved this study without requiring written informed consent from any of the patients because no data used in this study contained any personal information.

### Database

We used the health insurance claim database from the Japan Medical Data Center (JMDC), which contains medical data for patients using Employee Health Insurance, one of two major public insurance providers covering employees and their dependents. The JMDC was established in 2002 and is the largest medical database in Japan. The details of this database have been described elsewhere [24–26]. Briefly, the database records all individual medical claims from different hospitals, clinics, and pharmacies via a computer-aided postentry standardization method and an anonymous linkage system for patients using the same insurance provider. This database enables the aggregation of all claims for the same patient without duplicating medical claims.

The database includes data on age, sex, International Classification of Diseases 10th revision (ICD-10) diagnosis codes, the ICD-10 corresponding standard disease name master codes (referred as ICD-10 standard disease code), prescribed drugs, medical examinations, treatment, and the medical institution size, if medical records are available.

### Inclusion and exclusion criteria

This study included subjects who were registered in the JMDC database between 2005 and 2014 and for whom medical records for more than one year were available. All registered data in the database were included in the analysis. Subjects listed with any of the ICD-10 categories from E11 to E14 were extracted as candidates with DM. Among these subjects, those with a history of being prescribed any anti-DM drug, either an oral or injectable drug, were included in the analysis. Subjects satisfying any of the following conditions were excluded from the analysis. Subjects who terminated their Employee Health Insurance and for whom the subsequent insurance provider was unable to be identified. Subjects for whom any of the aforementioned DM-related ICD-10 information was not confirmed in the final record during the investigated period. We excluded patients without DM who were diagnosed with central retinopathy, macular edema, maculopathy, or cystoid macular edema because some of these individuals may have had DM undiagnosed.

### Definition of DME

Among subjects with DM, individuals with any of the following ICD10 subclassification disease names (ICD10 disease code) were categorized as having DME: diabetic maculopathy (E143), type 1 diabetic macula edema (E103), diabetic macula edema (E143), and type 2 diabetic maculopathy (E113).

### Comparison of DME-associated risk factors

We investigated risk factors associated with DME development by comparing the following two groups: (1) a group of patients with DME and a one-year claim record before the development of DME and (2) a group of patients without a DME diagnosis throughout the investigated period. Because we were unable to collect data regarding medical examination results for risk factors proposed in previous studies, including laboratory data, biological information, and genetic data, we focused on ICD-10 standard disease codes to identify risk factors associated with DME.

The current study compared ICD-10 standard disease codes for one year before DME development in a group of patients with DME and those for the most recent year in a group of patients without DME.

### Statistical analysis

For statistical analyses, the chi-square and Mann-Whitney U tests were employed to compare demographics between the DME and DME-free groups.

According to suggestions from two experts in the field of medical statistics, Dr. Hiroshi Yokomichi and Dr. Tatsuhiko Saigo, we applied the criteria listed below to limit ICD-10 codes for a proper analysis of these codes associated with the development of DME because the total number of ICD-10 codes was 6345, which may hinder accurate analysis of factors associated with DME development.

As the first step, ICD-10 codes satisfying the following two conditions were excluded: the total number of patients with that code was less than 0.1% of all enrolled subjects, and fewer than five subjects were diagnosed with that ICD-10 standard disease code. The chi-square test was utilized to identify associated disease codes, and codes with a *P* value of less than 0.05 were selected as significantly associated risk factors for DME in univariate analysis. Codes categorized as significant risk factors in univariate analysis were subjected to multivariate logistic regression analysis in addition to age and sex as risk factors, and codes with a *P* value less than 0.05 were considered significantly associated risk factors.

## Results

The total number of subjects registered in the database from 2005 to 2014 was 3,110,867. Of these, the total number of patients with DM was 66,923, with a mean age of 53.4 ± 11.0 years. Overall, 21,463 subjects had a DM-related ocular manifestation. After excluding subjects who did not satisfy the inclusion criteria, 17,403 individuals with a mean age of 55.7 ± 10.8 years were included in the analysis. Four hundred twenty subjects were diagnosed with DME, with a mean age of 55.2 ± 10.0 years, and the demographics of the enrolled subjects are listed in Table 1. DR was significantly more prevalent in males than females, but the incidence of DME development was not significantly different between males and females. Patients with and without DME visited medical institutions for diabetes treatment at frequencies of 3.2 times/year and 2.9 times/1 year, respectively, which was not a significant difference. The two groups also showed the same frequency of ophthalmological examinations (4.3 times/year). Among the 6345 ICD-10 standard disease codes, 86 were selected for investigating ICD-10 codes associated with the development of DME.

**Table 1:** Demographics of enrolled subjects

	Number	mean age ± SD (yrs.)	rate of male (%)		
All entry subjects	17,403	55.7 ± 10.4	65.0		
Type of DM (Type 1: Type 2: unclear)	(1710: 7026: 8667)				
				Developing rate of DME (%)	
Subjects with DME	420	55.7 ± 10.8	62.6	Male: 2.3	Female 2.6
Subjects without DME	16,983	56.2 ± 10.1	65.0		

*SD* standard deviation, *DME* diabetic macular edema

### Systemic factors associated with DME development: multivariate analysis

Systemic risk factors associated with DME development are shown in Table 2. Femoral head fractures showed the highest odds ratio (OROR, 7.04), followed by hyperlipidemia (OR 6.67) and impending abortion (OR 6.14). In contrast, four factors, chronic eczema (OR 0.12), ureterolithiasis (OR 0.13), hay fever (OR 0.13) and osteoarthritis of the knee (OR 0.55), were identified as factors that were negatively associated with DME development (Table 3).

**Table 2:** Systemic risk factors associated with DME development: multivariate analysis

ICD10 standard disease name	odds ratio	lower 95% CI	upper 95% CI	***P*** value
Femoral neck fracture	7.04	1.94	25.52	0.0030
Hyperlipidemia	6.67	2.26	19.65	0.0006
Impending abortion	6.14	2.35	16.07	0.0002
Ménière syndrome	5.20	1.44	18.77	0.0119
Cervical contusion	4.93	1.64	14.87	0.0046
Chest contusion	4.75	1.77	12.77	0.0020
Dysmenorrhea	4.72	1.62	13.75	0.0045
Arthritis	4.68	1.58	13.84	0.0052
Shoulder arthritis	3.84	1.54	9.57	0.0040
Diabetic ketoacidosis	3.28	1.62	6.65	0.0010
Vessel lumen mass	2.99	1.02	8.74	0.0456
Postoperative of Percutaneous Coronary angioplasty	2.96	1.01	8.61	0.0469
Lower leg skin ulcer	2.83	1.09	7.36	0.0328
Arrhythmia	2.82	1.20	6.65	0.0178
Diabetic nephropathy	2.52	1.56	4.07	0.0002
Proteinuria	1.90	1.05	3.41	0.0330

**Table 3:** Systemic suppressive factors associated with DME development: multivariate analysis

ICD10 standard disease name	odds ratio	lower 95% CI	upper 95% CI	***P*** value
Knee osteoarthritis	0.55	0.34	0.89	0.0145
Ureter lithiasis	0.13	0.02	0.96	0.0460
Hay fever	0.13	0.02	0.96	0.0450
Chronic eczema	0.12	0.02	0.89	0.0380

*ICD10* International Classification of Diseases 10th revision, *DME* diabetic macular edema, CI; confidential interval

### Ocular factors associated with DME development: multivariate analysis

Ten ocular risk factors associated with DME development were revealed (Table 4). Retinal vessel occlusion had the highest risk (OR 8.28), followed by eye movement disorder (OR 5.99). Intraocular lens insertion and posterior vitreous detachment were identified as significant factors in univariate analysis but not in multivariate analysis. No ICD-10 codes were identified as ocular factors having an inverse correlation with DME development.

**Table 4:** Ocular risk factors associated with DME development: multivariate analysis

ICD10 standard disease name	odds ratio	lower 95% CI	upper 95% CI	***P*** value
Retinal vessel occlusion	8.28	2.62	26.16	0.0003
Ocular movement disorder	5.99	2.01	17.82	0.0013
Accommodative paralysis	4.95	1.50	16.33	0.0087
Scleritis	4.63	1.27	16.88	0.0202
Corneal disease	4.01	1.08	14.94	0.0386
Ocular pain	3.21	1.08	9.61	0.0367
Retinal hemorrhage	2.85	1.48	5.47	0.0017
Vitreous hemorrhage	2.06	1.41	3.01	0.0002
Myopic astigmatism	1.56	1.25	1.96	0.0001
Conjunctivitis	1.35	1.05	1.73	0.0202

*ICD10* International Classification of Diseases 10th revision, *DME* diabetic macular edema, *CI* confidential interval

The results of univariate analysis for systemic and ocular factors associated with DME are summarized in Supplemental Tables 1 to 4.

## Discussion

Although the global incidence of DM is increasing, that of DR is reported to be decreasing [27]. This finding might be explained by improvements in the control of systemic risk factors in patients with DM. DM-related severe ocular complications are one of the main causes of blindness worldwide. According to a recent study, DME is causing an increasing number of visual impairments in patients with DM [22]. DME, which does not result in total blindness but in severe vision loss, may instead be the main DM-associated severe ocular complication. In addition, advances in optical coherence tomography technology have enabled the identification of DME much more precisely and less invasively than before. Some previous studies have investigated factors associated with DME. However, many of these studies employed a cross-sectional design [8, 22, 23, 28–31]. Although several studies have investigated factors associated with DME development, [2, 14, 32–35] associations between concomitant and systemic diseases with DME were not reported. In the present study, we investigated factors associated with DME development among more than 6000 ICD-10 standard disease codes using a large claim dataset in Japan. Many of the systemic factors significantly associated with the development of DME are considered to be related to the presence of severe metabolic impairment, including hyperlipidemia, diabetic ketoacidosis, vessel lumen mass, postoperative percutaneous coronary angioplasty, lower leg skin ulcer, arrhythmia, diabetic nephropathy, and proteinuria. Impairment of microvasculature in the retina results in breakdown of the retinal pigment epithelial barrier, which is one of the key steps in DME development. Many systemic factors, including hyperglycemia, inflammation, and vascular endothelial dysfunction, are associated with DM-related microvasculature damage. Accordingly, there is a clear relationship between systemic microvasculature damage and ocular complications. Yamamoto et al. reported that diabetes, proteinuria and glomerular filtration rate were associated with higher risks of diabetic eye diseases, including DME [36].

DM has been identified as an important risk factor for osteoporosis-associated fracture [37, 38]. Among female subjects with type 1 DM, diabetic ketoacidosis in pregnancy can result in impending abortion. Because DM sometimes results in peripheral vestibular damage and predicts a poor prognosis of typical vestibular pathologies, some patients develop benign paroxysmal positional vertigo, which is one symptom of Ménière syndrome. Poor visual function due to DME may contribute to increased risk of falls, resulting in cervical and chest contusions. Furthermore, female patients with DM are more likely to report very irregular menstrual cycles [39]. Although the present study identified arthritis as a risk factor for developing DME, Tentolouris et al. found a negative correlation between arthritis and DM [40]. Regardless, the impact of DM on the incidence of rheumatoid arthritis is not well established.

The present study also revealed some systemic factors that were negatively associated with the development of DME. Some previous studies reported a significantly higher prevalence of primary osteoarthritis among subjects with DM than among those without DM, as well as significant associations with glycemic control and the duration of diabetes. Obesity may be associated with the onset of osteoarthritis, [41–43] though we are unsure why our study showed a negative impact of osteoarthritis on DME. According to Taylor et al., diabetes may increase the risk of kidney stone formation by altering the composition of the urine, and insulin resistance may play a role in stone formation [44].

Moreover, several systemic diseases and ocular diseases that have not been reported to be related to DME development were identified. Interestingly, some factors were identified as negative factors. An explanation for the finding that hay fever and chronic eczema were significantly and negatively associated with DME development may be impairment of autoimmune function, as DM deteriorates autoimmune function, and a significantly lower prevalence of allergic rhinitis has been observed in subjects with metabolic syndrome, high blood pressure, or impaired fasting glucose levels [45].

Many previous studies have focused on the effects of DM on pathological conditions and disorders, whereas few have investigated factors associated with the development of DME in a large sample. We were unable to precisely investigate the status of glycemic control and severity of DM complications in the present study, and further studies are thus necessary to clarify these points.Although some significantly associated systemic factors identified in the current study are consistent with previous reports, some of them have never been reported to be associated with the development of DME. In addition to diseases found to act as risk factors to date, such as renal and circulatory disorders, orthopedic and dermatological diseases were identified to be associated with the development of DME, which may be because the current study included more than 6000 diseases in the analysis. Furthermore, some factors were found to be negatively associated with DME. Currently, information about the mechanisms of these factors in DME development is limited, and these data must be confirmed in further investigations.

Previous papers have reported some ocular factors associated with DME development, including DR severity, [2, 32, 46] cataract surgery, [33] and ocular inflammation, [35] consistent with the current results. The present study also revealed some new ocular factors associated with DME development, some of which are related to systemic and ocular DM complications. As possible explanations for these associations, ocular movement disorder and accommodative paralysis or corneal disease and ocular pain may be related to microvasculature destruction or DM-associated loss of tear-film stability, respectively. Additionally, scleritis and conjunctivitis may be related to DM-induced microinflammation. Retinal vessel occlusion showed the highest OR and was selected as a disease often observed in subjects with retinal circulation disorders and severe diabetic retinopathy. Moreover, eye movement disorder and accommodation paralysis are strongly associated with DME development. Further studies are necessary to clarify the mechanism between DME and systemic or ocular risk factors.

This study also has several limitations. Because the subjects were limited to social insurance subscribers and because subscribers of national health insurance, another major insurance provider in Japan, was not considered, the target sample may be biased. National health insurance is managed by each municipality, increasing the difficulty of integrating and collecting data; hence, these data were not included in the study. As subjects in JMDC constitute employees and their dependents, it is possible that a significant number of senior citizens, whose prevalence of DM could be higher than that in JMDC, were not included in the database. National health insurance members must also be considered in the future. The use of diagnosis codes of claims data as diagnostic criteria also has several problems. First, the accuracy of the diagnosis is not necessarily high. The currently used database may include subjects who have not been medically diagnosed with DM to obtain reimbursement from health insurance. Therefore, only patients who were prescribed anti-DM drugs were included in this study. An investigation of whether some reported risk factors are associated with DME development was impossible because the current database does not contain information about certain factors, including hemoglobin A1c levels [14] and the severity of DR [2, 32, 46]. Genetic factors have also been reported to contribute to DME development, such as genes related to VEGF and erythropoietin [47–51]. Unfortunately, claims data do not contain genetic information. In this study, we worked with epidemiological statisticians to accurately detect risk factors associated with DME development from many disease categories, but it was difficult to completely exclude the influence of confounding factors. Because no accurate information regarding the type of DM was available for approximately half of all subjects, it was impossible to compare risk factors between type 1 DME and type 2 DME. We excluded some diseases due to a small number of patients, which may have involved factors for which we could not detect an association. Inconsistencies between previous reports and the current study may also be partially due to differences in the statistical methods applied. Previous studies have reported differences between DR- and DME-associated factors. In this study, we focused on factors associated with the development of DME. In the future, it will be necessary to examine factors associated with the development of DMR and DME using the same database.

## Conclusions

In this study, we clarified systemic and ocular diseases associated with DME development in Japan. Notably, many patients with DM do not undergo periodic eye examinations. Based on the results, factors associated with DME development should be closely monitored. Because DR is may be asymptomatic during the period in which laser photocoagulation should be applied, asymptomatic individuals should be screened to minimize the risk of vision loss. As the number of DME patients is expected to increase, further studies on the early detection and prevention of DME development are needed.

## Supplementary information

**Additional file 1: Supplemental Table 1** Systemic risk factors associated with DME development: univariate analysis. (ICD10; International Classification of Diseases 10th revision, DME; diabetic macular edema, CI; confidence interval).

**Additional file 2: Supplemental Table 2** Systemic factors that suppressed DME development: univariate analysis. (ICD10; International Classification of Diseases 10th revision, DME; diabetic macular edema, CI; confidence interval).

**Additional file 3: Supplemental Table 3** Ocular risk factors associated with DME development: univariate analysis. (ICD10; International Classification of Diseases 10th revision, DME; diabetic macular edema, CI; confidence interval).

**Additional file 4: Supplemental Table 4** Ocular factors that suppressed DME development: univariate analysis. (ICD10; International Classification of Diseases 10th revision, DME; diabetic macular edema, CI; confidence interval).

## Data Availability

The data that support the findings of this study are available from the JMDC, but restrictions apply with regard to the availability of these data, which were used under license for the current study, and thus are not publicly available. The data are, however, available from the authors upon reasonable request and with permission of the JMDC.

## Abbreviations

DM: Diabetic mellitus
DME: Diabetic macular edema
DR: Diabetic retinopathy
PDR: Proliferative diabetic retinopathy
CI: Confidence interval
ICD-10: International Classification of Diseases 10th revision
JMDC: Japan Medical Data Center
OR: Odds ratio
VEGF: Vascular endothelial growth factor
