# Supplementary material for: Systemic and ocular diseases associated with the development of diabetic macular edema among Japanese patients with diabetes mellitus
Source: BMC Ophthalmol. 2020 Jul 29;20:309. doi: 10.1186/s12886-020-01578-8 (PMC7392833; doi:10.1186/s12886-020-01578-8)
Supplement: Supplementary file 1 — Additional file 1: Supplemental Table 1 Systemic risk factors associated with DME development: univariate analysis. (ICD10; International Classification of Diseases 10th revision, DME; diabetic macular edema, CI; confidence interval). [file 12886_2020_1578_MOESM1_ESM.docx]

Supplemental Table 1 Systemic risk factors for DME development: univariate analysis

| **ICD10 standard disease name** | **Odd ratio** | **Lower 95% CI** | **Upper 95% CI** | **P value** |
| --- | --- | --- | --- | --- |
| Femoral neck fracture | 7.04 | 1.94 | 25.52 | 0.0013 |
| High lipidemia | 6.67 | 2.26 | 19.65 | 0.0003 |
| Imminent abortion | 6.14 | 2.35 | 16.07 | 0.0001 |
| Ménière syndrome | 5.20 | 1.44 | 18.77 | 0.0009 |
| Cervical contusion | 4.93 | 1.64 | 14.87 | 0.0026 |
| Chest contusion | 4.75 | 1.77 | 12.77 | 0.0009 |
| Dysmenorrhea | 4.72 | 1.62 | 13.75 | 0.0002 |
| Arthritis | 4.68 | 1.58 | 13.84 | 0.0054 |
| Hypoproteinemia | 4.68 | 1.58 | 13.84 | 0.0054 |
| Shoulder arthritis | 3.84 | 1.54 | 9.57 | 0.0002 |
| Diabetic ketoacidosis | 3.28 | 1.62 | 6.65 | <0.0001 |
| Lumen mass | 2.99 | 1.02 | 8.74 | 0.0233 |
| Postoperative of Percutaneous coronary angioplasty | 2.96 | 1.01 | 8.61 | 0.0209 |
| Lower leg skin ulcer | 2.83 | 1.09 | 7.36 | <0.0001 |
| Arrhythmia | 2.82 | 1.20 | 6.65 | 0.0006 |
| Skin defect | 2.77 | 0.84 | 9.11 | 0.0003 |
| Shock | 2.53 | 0.56 | 11.52 | 0.0067 |
| Diabetic nephropathy | 2.52 | 1.56 | 4.07 | <0.0001 |
| Numbness | 2.40 | 0.78 | 7.40 | 0.0039 |
| Foot osteomyelitis | 2.18 | 0.53 | 9.01 | <0.0001 |
| Proteinuria | 1.90 | 1.05 | 3.41 | 0.0288 |
| Hemorrhagic anemia | 1.71 | 0.41 | 7.12 | 0.0148 |
| Diabetic gangrene | 1.43 | 0.63 | 3.28 | <0.0001 |
| Sepsis | 1.43 | 0.53 | 3.84 | 0.0069 |
| Congestive heart failure | 1.30 | 0.79 | 2.14 | 0.0008 |
| Diabetic peripheral nephropathy | 1.28 | 0.94 | 1.75 | 0.0016 |
| Renal failure | 1.23 | 0.74 | 2.05 | 0.0001 |
| Hypertension | 1.19 | 0.95 | 1.47 | 0.0017 |

(ICD10; International Classification of Diseases 10th revision, DME; diabetic macular edema, CI; confidential interval)
