# Supplementary material for: Systemic and ocular diseases associated with the development of diabetic macular edema among Japanese patients with diabetes mellitus
Source: BMC Ophthalmol. 2020 Jul 29;20:309. doi: 10.1186/s12886-020-01578-8 (PMC7392833; doi:10.1186/s12886-020-01578-8)
Supplement: Supplementary file 2 — Additional file 2: Supplemental Table 2 Systemic factors that suppressed DME development: univariate analysis. (ICD10; International Classification of Diseases 10th revision, DME; diabetic macular edema, CI; confidence interval). [file 12886_2020_1578_MOESM2_ESM.docx]

**Supplemental Table 2 Systemic suppressive factors of DME development: univariate analysis**

| **ICD10 standard disease name** | **Odd ratio** | **Lower 95% CI** | **Upper 95% CI** | **P value** |
| --- | --- | --- | --- | --- |
| Lateral epicondylitis | 0.00 | 0.00 | - | 0.0297 |
| Chronic eczema | 0.12 | 0.02 | 0.89 | 0.0200 |
| Hay fever | 0.13 | 0.02 | 0.96 | 0.0103 |
| Ureter lithiasis | 0.13 | 0.02 | 0.96 | 0.0115 |
| Earwax plug | 0.34 | 0.11 | 1.06 | 0.0241 |
| Insomnia | 0.52 | 0.37 | 0.72 | 0.0191 |
| Osteoarthritis of the knee | 0.55 | 0.34 | 0.89 | 0.0195 |
| Renal anemia | 0.65 | 0.31 | 1.36 | 0.0017 |
| Fatty liver | 0.713 | 0.50 | 1.01 | 0.0189 |
| Secondary hyperparathyroidism | 0.92 | 0.41 | 2.07 | 0.0148 |
| Hyperkalemia | 0.96 | 0.50 | 1.84 | 0.0101 |

(ICD10; International Classification of Diseases 10th revision, DME; diabetic macular edema, CI; confidential interval)
