# Supplementary material for: Systemic and ocular diseases associated with the development of diabetic macular edema among Japanese patients with diabetes mellitus
Source: BMC Ophthalmol. 2020 Jul 29;20:309. doi: 10.1186/s12886-020-01578-8 (PMC7392833; doi:10.1186/s12886-020-01578-8)
Supplement: Supplementary file 3 — Additional file 3: Supplemental Table 3 Ocular risk factors associated with DME development: univariate analysis. (ICD10; International Classification of Diseases 10th revision, DME; diabetic macular edema, CI; confidence interval). [file 12886_2020_1578_MOESM3_ESM.docx]

**Supplemental Table 3 Local risk factors of DME development: univariate analysis**

| **ICD10 standard disease name** | **odd ratio** | **Lower 95% CI** | **Upper 95% CI** | **P value** |
| --- | --- | --- | --- | --- |
| Retinal vessel occlusion | 8.28 | 2.62 | 26.16 | <0.0001 |
| Eye movement disorder | 5.99 | 2.01 | 17.82 | <0.0001 |
| Accommodative paralysis | 4.95 | 1.50 | 16.33 | <0.0001 |
| Scleritis | 4.63 | 1.27 | 16.88 | 0.0083 |
| Corneal disease | 4.01 | 1.08 | 14.94 | 0.0013 |
| Ocular pain | 3.21 | 1.08 | 9.61 | 0.0007 |
| Retinal hemorrhage | 2.85 | 1.48 | 5.47 | <0.0001 |
| Uveitis | 2.73 | 1.75 | 4.26 | <0.0001 |
| Vitreous hemorrhage | 2.06 | 1.41 | 3.01 | <0.0001 |
| Cataract | 1.89 | 1.54 | 2.32 | <0.0001 |
| Myopic astigmatism | 1.56 | 1.25 | 1.96 | <0.0001 |
| Conjunctivitis | 1.35 | 1.05 | 1.73 | <0.0001 |
| Ocular hypertension | 1.16 | 0.77 | 1.75 | 0.0154 |

(ICD10; International Classification of Diseases 10th revision, DME; diabetic macular edema, CI; confidential interval)
