# Supplementary material for: Systemic and ocular diseases associated with the development of diabetic macular edema among Japanese patients with diabetes mellitus
Source: BMC Ophthalmol. 2020 Jul 29;20:309. doi: 10.1186/s12886-020-01578-8 (PMC7392833; doi:10.1186/s12886-020-01578-8)
Supplement: Supplementary file 4 — Additional file 4: Supplemental Table 4 Ocular factors that suppressed DME development: univariate analysis. (ICD10; International Classification of Diseases 10th revision, DME; diabetic macular edema, CI; confidence interval). [file 12886_2020_1578_MOESM4_ESM.docx]

**Supplemental Table 4 Local suppressive factors of DME development: univariate analysis**

| **ICD10 standard disease name** | **odd ratio** | **Lower 95% CI** | **upper 95% CI** | **P value** |
| --- | --- | --- | --- | --- |
| Posterior vitreous detachment | 6.07^-08^ | 0.00 | NA | 0.0176 |
| Neovascular glaucoma | 0.68 | 0.29 | 1.60 | 0.0224 |
| Intraocular lens inserted eye | 0.89 | 0.66 | 1.19 | 0.0005 |

(ICD10; International Classification of Diseases 10th revision, DME; diabetic macular edema, CI; confidential interval)
